# Social Capital From Professional Engineering Organizations and the Persistence of Women and Underrepresented Minority Undergraduates

**DOI:** 10.3389/fsoc.2021.671856

**Published:** 2021-05-31

**Authors:** Chrystal A. S. Smith, Hesborn Wao, Gladis Kersaint, Rebecca Campbell-Montalvo, Phyllis Gray-Ray, Ellen Puccia, Julie P. Martin, Reginald Lee, John Skvoretz, George MacDonald

**Affiliations:** ^1^ Department of Anthropology, University of Connecticut, Storrs, CT, United States; ^2^ Department of Curriculum and Instruction, Neag School of Education, University of Connecticut, Storrs, CT, United States; ^3^ Department of Sociology and Criminal Justice, Florida A&M University, Tallahassee, FL, United States; ^4^ Beta Research Associates, Palmetto, FL, United States; ^5^ Department of Engineering Education, The Ohio State University, Columbus, OH, United States; ^6^ Center for Research, Evaluation, Assessment, Measurement, College of Education, University of South Florida, Tampa, FL, United States; ^7^ Department of Sociology, University of South Florida, Tampa, FL, United States; ^8^ The MacDonald Research Institute, Wesley Chapel, FL, United States

**Keywords:** STEM degree persistence, equity, engineering education, professional engineering organizations, social capital

## Abstract

Professional engineering organizations (PEOs) have the potential to provide women and underrepresented and minoritized (URM) students with social capital (i.e., resources gained from relationships) that aids their persistence in their engineering undergraduate programs and into the workforce. We hypothesize that women and URM students engineering students who participate in PEOs are more likely to persist in their engineering major and that PEOs contribute to their persistence by providing them access to insider information that supports their persistence. Each year for five years we administered surveys with closed- and open-ended items to examine the association between participating in PEOs and the persistence of a cohort of engineering majors from 11 diverse universities. We used logistic regression and thematic analysis to analyze the data. URM students who participated in PEOs and other engineering related activities were more likely to persist to the second year than URM students who did not (adjusted odds ratio = 2.18, CI: 1.09, 4.37). Students reported that PEOs contributed to their persistence by enabling them to network, reduce gender and race/ethnic isolation, and access professional resources. URM students should be encouraged to participate in PEOs beginning in their first year to increase their integration in their major, which we have found to increase their persistence.

## Introduction

The culture of engineering undergraduate degree programs is often unwelcoming and exclusionary for women and underrepresented and minoritized (URM) students, who are often subjected to overt sexism, racism, discrimination, stereotyping, and isolation (May and Chubin, 2003; Brown et al., 2005; McGee and Martin, 2011; Geisinger and Raj Raman, 2013; Seron et al., 2015; McGee, 2016; McGee, 2020). According to the National Science Foundation (NSF 2019), URM students in science, technology, engineering and mathematics (STEM) include Black/African American, Latinx, and American Indian and Alaskan Native men and women. The hostile climate of engineering programs and the feeling of not belonging in these programs are the main reasons that students identify for switching to non-engineering majors (as well as non-STEM majors) before graduation (Seymour and Hewitt, 1997; Tyson et al., 2007; Griffith, 2010; Hill et al., 2010; Ohland et al., 2011; Marra et al., 2012; Meyer and Marx, 2014; Rainey et al., 2018; Fink et al., 2020). This negative academic climate is a threat to efforts to make the STEM workforce more diverse, equitable, and inclusive.

Student involvement research has resulted in inconsistent findings about the benefits of student participation in PEOs and few are quantitative or mixed methods studies that examine the association with persistence. For example (Wilson et al., 2014), found that student participation in professional societies and other academic activities was positively associated with “self-efficacy and academic emotional engagement,” but students who participated in women and minority organizations had “lower academic emotional engagement” than their counterparts who did not. They concluded that this lower emotional engagement is a coping mechanism where students practice detachment to curricula that has been traditionally shaped around the needs of the dominant group. Other research found students did not identify their participation in PEOs as important for their academic engagement (Allendoerfer et al., 2012) and found that students had levels of activity with their jobs and sports and less activity in PEOs (Simmons et al., 2018). In contrast, other studies have found that student participation in engineering PEOs, including the National Society of Black Engineers (NSBE) and the Society of Hispanic Professional Engineers (SHPE) provides social capital, supportive environments, and cultural enclaves that help students combat isolation, leading to greater on campus integration (Daily et al., 2007; Strauss and Terenzini, 2007; Martin et al., 2016; Ross and McGrade, 2016; Revelo and Baber, 2018). Several student involvement studies reveal that participation in and integration of co-curricular or extracurricular activities is one means of increasing persistence (Tinto, 1998; Astin, 1999; Berger and Milem, 1999; May and Chubin, 2003).

To bolster the methodological approaches used in previous qualitative studies, we use a mixed methods approach with a diverse student data set to investigate how women and URM students’ persistence may be affected by the social capital acquired from their participation in PEOs. Social capital refers to the individuals in a person’s social network and the resources that can be accessed through that network (Lin, 2001). For engineering students, such resources include access to professional role models and potential employers, opportunities to serve as leaders and develop leadership skills, enculturation into professional norms, and access to a network of like-minded peers who provide insight about which classes may be better to take, copies of past exams and other study materials (Smith et al., 2015).

Building upon Lin (2001), we focus on the relationships that student members cultivate with others in PEOs as a primary source of the social capital. Previously, we have differentiated between “participatory social capital”, the capital gained by participants through the relationships they gain by participating in organizations that facilitate such networking (i.e. professional engineering organizations), and “network-based” social capital, the capital students access through social networks they have through their life and matriculation in their program more generally (i.e. family, professors) (Skvoretz et al., 2020; Puccia et al., 2021). Both types of social capital provide support, including that linked to direct forms of support to continue in STEM (i.e. travel awards, advice about what classes to take) as well as emotional support (i.e. advice about how to respond to negative treatment by others in STEM) (Puccia et al., 2021; Segarra et al., 2020). This research extends these prior findings by examining PEO participation rates across groups, how participation in various women- and race/ethnicity-focused PEOs in their first year of their engineering degree program affects women and URM student’s persistence to their fifth year, as well as identifying the specific mechanisms through which student gain social capital through these organizations.

## Background

### Persistence of Undergraduate Students

The persistence of women and URM students in engineering can be understood within the broader literature about the persistence of all undergraduate students. Astin’s (1999) student involvement theory and (Tinto’s 1998) theory of student departure assert that higher levels of student involvement and integration in campus life lead to improved student learning outcomes and persistence. Student involvement, according to Astin (1999), describes a student who is not only succeeding academically, but also “spends much time on campus, participates in student organizations, and interacts frequently with faculty members and other students” (p. 518).

Similarly, Tinto (1998) describes student involvement as “academic and social integration.” Academic integration includes academic achievement and interaction with faculty and peers. Social integration primarily refers to the extent to which students socialize with peers and have feelings of fitting in. Tinto (1998) contends that this integration is a main influence on student persistence and is particularly crucial during the first year of college, when the attrition rate is the highest. Students must be motivated to commit time and become active participants in their academic and social college experiences (Tinto, 1998; Astin, 1999).

Scholarship on the persistence of women and URM students must attend to the different academic climate they experience compared to their majority peers, particularly when considering the potential role of PEOs in mitigating the effects of hostile climates on persistence. Often, members of the dominant group (i.e., White, Asian) are not aware of how race or gender affects other students’ experiences and outcomes in undergraduate STEM programs (Dancy et al., 2020). Specifically, cultural ideologies associate men and masculinity with technical engineering skills (Dryburgh, 1999; Faulkner, 2000; Faulkner, 2009) and can result in interpersonal student behavior that restricts women’s access to technical activities in lab and other engineering settings.

Likewise, STEM programs often prioritize individualistic and competitive cultures, which are attributes frequently associated with majority groups (Faulkner, 2009; Ong,et al., 2018; Secules et al., 2018; McGee, 2020). This results in mismatches between academic program cultures and the values of URM students, especially women of color, who may feel like they do not fit in STEM programs (Rainey et al., 2018) and report that their STEM experiences are explicitly impacted by race and gender (Dancy et al., 2020). Black students are disproportionately affected by their STEM programs' climates, given the societal pervasiveness of anti-Black microaggressions (Lee et al., 2020). Because the relationship between fitting into unwelcome academic climates and persistence is strong (Marra et al., 2012; Meyer and Marx, 2014; Rainey et al., 2018; Fink et al., 2020), investigations into how students gain social capital through PEOs to deal with poor academic climate are imperative.

### Influence of Professional Engineering Organizations

Student chapters of PEOs are mechanisms by which engineering students can become integrated both academically and socially. Women- and race/ethnicity-focused PEOs, such as the Society of Women Engineers (SWE), the National Society of Black Engineers (NSBE), and the Society of Hispanic Professional Engineers (SHPE), contribute to students’ identity development as engineers, to their persistence, and to their success in their engineering studies and subsequent careers (Goodman et al., 2002; Daily et al., 2007; Martin et al., 2016; Ross and McGrade, 2016; Revelo and Baber, 2018). They are often welcoming environments that reduce students’ gender/ethnic isolation. They can also break down cultural barriers to integration in programs in which white men are the majority. In addition, they often serve as safe spaces where women and URM students can rebuild their confidence and motivation when they struggle academically (Meyer and Marx, 2014).

PEOs can provide women and URM students with familiar cultural environments that prioritize serving the community and collectivism over individualism and the academic competition that drives engineering programs (Seymour and Hewitt, 1997; Martin et al., 2016). These women- and race/ethnicity-focused PEOs can also offer women and URM students a “culture of support” and “motivation to succeed,” which is associated with student persistence (Grandy, 1998; Suresh, 2006). Goodman and colleagues (2002) assert that by participating in PEOs and other support activities, women can build networks that create a community that makes them feel less isolated. They found that two-thirds of the women in their sample participated in PEOs to socialize with other women in engineering, and two-fifths of women did so because of the supportive environment. Daily and colleagues (2007) found that through its social network/connections, student leadership opportunities, and other activities (e.g., attending professional conferences for career fairs), NSBE creates a culture referred to as “luv.” By participating in this culture, Black students acquire social capital that supports academic achievement and retention in their engineering programs. When considered collectively, the literature provides robust support for the vital role that PEOs, especially those that are gender- and race/ethnicity focused, can play in the persistence, especially for women and URM students.

## Career Advancement

For women and URM students, PEOs, particularly women- and race/ethnicity-focused PEOs (e.g., SWE, NSBE, and SHPE) are invaluable sources of social capital. This social capital is specifically intended to increase women and URM students’ academic achievement and advance their careers, thus broadening their participation in engineering. To further these goals, women- and race/ethnicity-focused PEOs provide resource-rich environments in which collegiate members can access and activate insider knowledge and resources that would not be available to them otherwise. Because they are viewed as outsiders in engineering, women and URM students also participate in social interactions that support their integration and confidence in engineering. To determine how participating in PEOs affect women and URM students’ engineering persistence, we investigated the following two research questions:

1. What is the relationship between participation in PEOs in year one and whether women and URM students persisted in their engineering degree programs in later years?
2. How do women and URM students believe that participation in PEOs contributed to their persistence?

The answer to research question 1 (RQ1) informs the association between participating in PEOs and persistence in engineering. The answers to research question 2 (RQ2) offer insight into the mechanisms through which social capital can influence persistence.

## Conceptual Framework

Originally (Bourdieu 1986), proposed social capital as primarily “connections” or access to a well-established network of useful relationships and material resources that benefit group members. Since then, social capital has been conceptualized in a variety of ways by numerous scholars (Coleman, 1988; Portes, 1998; Lin, 2001; Adler and Kwon, 2002; Bandiera et al., 2008; Korte and Lin, 2011). For example, Coleman (1988) described social capital as a function within societal structure, a group asset embedded in an individual’s relationships. In contrast, Lin (2001) argues that the focus should be on the resources of individuals that benefit the group. Other scholars suggest that social capital in postsecondary education reproduces class and gender inequality (Holland and Eisenhart, 1990).

To study how an individual’s social network contributes to their educational success, we adhere to (Lin’s 2001) conceptualization that social capital “is captured in social relations and that its capture evokes structural constraints and opportunities as well as actions and choices on the part of the actors” (p. 3). Lin’s conceptualization acknowledges that ascribed characteristics such as gender and race/ethnicity often create differences in access to social capital. Lin also asserts that individuals can achieve goals through activating social capital in “purposive actions” (2001, p. 60). Thus, our research examines students’ social networks and the individuals in those social networks who influence their persistence in engineering. We also investigate how accessing and activating the social capital available in their social networks contribute to their persistence in engineering. Martin (2015) highlights the potential of “resource-rich networks” that do “not necessitate students knowingly mobilizing resources” because they “receive information and resources in routine exchanges” with the faculty and administration in engineering programs (p. 1180).

Because engineering is a field that white men have traditionally dominated, it has gained a reputation as a “closed club” (Ohland et al., 2011) where women and URM students are often treated like outsiders (Tate and Linn, 2005). Therefore, they are less likely to have the social relations (i.e. social networks) with individuals who are “insiders” and know about the “informal” pathways and resources that lead to success in engineering and other STEM fields (Seymour, 1999; Stevens et al., 2008). By cultivating these relationships, students acquire social capital that aids their persistence to degree attainment (Atman et al., 2008; Stevens et al., 2008; Shapiro and Sax, 2011). These relationships can further help women and URM students navigate the generally unwelcoming academic climate by creating culturally familiar and welcoming spaces that contribute to their persistence.

## Methods

### Instrumentation

To answer the research questions, we developed five surveys that were IRB-approved and administered annually to a cohort of engineering undergraduates, beginning at the end of their first year of their program. The first survey measured the social capital students brought from high school and other pre-college experiences into their engineering programs. The four subsequent surveys, parallel in structure, measured the social capital acquired while students were enrolled in their engineering programs. Social capital survey items asked students (egos) about their participation in PEOs, professional societies, and other engineering-related activities and programs. Students were also asked to identify individuals who advised them to participate in PEOs and other activities, the extent of their participation, and how their participation contributed to their persistence (with this last item being the only open-ended item).

We created social capital items from the activities and resources that 31 stakeholders identified as beneficial for undergraduate success in engineering programs (Smith et al., 2015). These stakeholders were engineering faculty, advisors, graduate students, and undergraduates who participated in a *free listing* exercise, an anthropological qualitative research method. Free listing assumes that individuals 1) with extensive knowledge will provide more responses than those with less knowledge, 2) will list most familiar and meaningful responses first, and 3) will provide responses that reflect their local cultural knowledge (Weller and Romney, 1988).

The first (S1) and second (S2) surveys were pilot tested with a diverse sample of 30 engineering undergraduates who were not part of the study to refine each survey and increase its validity. We also conducted the *think-aloud* exercise (Smith et al., 2015) with S1 and S2, a verbal cognitive validation protocol, with a diverse sample of nine engineering undergraduates who were not part of the study (Martin et al., 2011). The students were asked to evaluate the survey, comment on item clarity, and suggest how unclear questions could be revised to be more explicit. Students read the questions and answers aloud while taking the online survey in a researcher’s office. Researchers who engaged in this process observed the students’ body language and listened to their comments as they cognitively processed their responses aloud. Feedback from the think-aloud exercise, which indicated several cognitive and minor structural issues, was used to revise the survey. Both refined surveys were then reliability-tested for internal consistency with 100 engineering undergraduates who were also not part of this study. Once we were confident in the reliability and validity of the items, the surveys were finalized. The third (S3), fourth (S4), and fifth (S5) surveys were modified versions of S2 that inquired about the students’ experiences in the previous year so they were not tested and validated individually.

### Study Sample and Data Collection

In spring 2015, engineering undergraduates were recruited from a population of all first-year students enrolled in the engineering programs at 11 universities located in three states and one U.S. territory. These engineering programs represent diverse learning contexts: five predominantly white institution (PWIs), three Hispanic-Serving Institutions (HSIs), two private PWIs, and one Historically Black College/University (HBCU). The engineering programs at these 11 universities had a total enrollment of approximately 5,854 first-year students in fall 2014. Each engineering program emailed their first-year students encouraging them to participate in our study. We sent three reminder recruitment emails with the link to S1 to all of these students over a period of two months.

Overall, 2,186 students of all genders and race/ethnicities completed S1. This sample represents a 37% response rate of the total enrollment in engineering programs at all participating universities. Students’ statuses as an enrolled first-year engineering major were verified by an institutional representative from the engineering program at the 11 universities, as explained to students on the informed consent page presented before the survey items. We administered subsequent surveys (S2, S3, S4, and S5) to students who reported that they were still enrolled as engineering majors in the previous survey only. Each student who responded to S1 was sent a recruitment email with an assigned identifier that allowed us to link their responses across the five surveys. Over the course of the study, we lost 850 students (40% of the initial 2,186 sample) to attrition. The biggest loss was between S1 and S2, when 432 of S1 respondents failed to take S2. The number of students lost to attrition declined each year as the study progressed. However, there was a slight uptick in attrition in the response to S5 likely due to natural disasters that occurred during the period the survey was administered. Eighty-four respondents were excluded due to later determination based on their survey responses that they had never intended to pursue engineering as a major, specifically these students were computer science majors, which is not classified as an engineering field by NSF or the National Center for Education Statistics.

Table 1 presents the survey response and persistence data for the students in the sample after the administration of each survey. We administered S1 in spring 2015, and by then some students may have already switched out of engineering to non-STEM majors. We refer to these students as “switchers.” This might explain the higher graduation rate of the students in our sample compared to the national rates (49% in our study graduated within five years vs. the 33% nationally who graduated within four years, according to figures from 2011) (American Society for Engineering Education, 2017). However, we recognize that most statistics are on engineering degree attainment are based upon four years of data, whereas we have five years available. Alternatively, it may be that students who are more likely to stay in engineering decided to take the survey. Students who reported that they were no longer enrolled at university are considered “leavers.”

**TABLE 1:** Number of Respondents, Broken down by Enrollment Status.

Survey	Respondents	Still enrolled	Eng. Grads	Switchers	Leavers	Non-eng. Grads
S1	1,252	1,252	–	–	–	
S2	1,252	1,003	1	232	16	–
S3	1,003	947	7	36	13	–
S4	947	883	36	17	11	–
S5	883	235	616	11	17	4

Note: This table excludes non-engineering majors and those lost to attrition; Eng. Grads = graduated with an engineering degree; Switchers = changed to a non-engineering major; leavers = left university; Non-Eng Grads = graduated with non-engineering degree.

### Data Source and Analysis

The specific survey items used to answer the research questions are presented in Table 2. The gender (binary: woman vs. man) and race/ethnicity data (eight categories) collected in S1 were used for intersectional analysis with the exception of eight students who did not provide their race/ethnicity data in S1 (we used the information provided in S2).

**TABLE 2:** Quantitative and Qualitative Data Sources.

Research Question	Quantitative and qualitative data source
Gender and race/Ethnicity	Survey 1 Items
	•Gender: Female; male
	•Race/ethnicity: American indian/Alaska native; asian (asian indian, Chinese, Filipino, Japanese, Korean, Vietnamese, other asian______); black/Black; hispanic (cuban, mexican, mexican american, puerto rican, other hispanic or latino origin_______); native Hawaiian/Other pacific islander; middle eastern/North african/Arab; white; other ethnicity________
PEO participation	Survey 2 Items
	In my first year as an engineering major, I participated in the following organizations/societies (select all that apply): 1) honor societies (e.g., tau beta pi [TBP]), alpha pi mu [APM]); 2) industry or discipline specific societies; 3) mexican americans in engineering and science (MAES) (e.g., bohique); 4) NSBE; 5) SHPE; 6) SWE; 7) other organizations/societies (to be specified); and 8) none
Research Question 1	Survey 2–5 Items
	•I am currently enrolled as an undergraduate engineering student: 1) yes; 2) no, I switched to a non-engineering major; 3) no, I am no longer enrolled at any university/college; 4) I graduated with my engineering degree; 5) I graduated with a non-engineering degree
	Survey 5 Items
	•In my first year as an engineering major, I participated in the [*insert identified organization/society*] because: 1) I was advised to participate by the [*insert alter in social network*] who most influenced me; 2) I was advised to participate by the other person(s) who influenced me; 3) my department/college assigned me to this activity; 4) and I decided on my own to participate
	•On a 4.0 scale, my grade point average at my current university is: ________
	•In my first year as an engineering major, my employment status was (select all that apply): 1) did not work; 2) federal work study; 3) full-time employee; 4) part-time employee; and 5) summer/seasonal employment only
	•In my first year as an engineering major, the estimated number of hours that I spent on my studies outside of class meeting times (i.e., assigned projects, studying individually and with study groups) in a typical week was 1) <15 h; 2) 15–19 h; 3) 20–24 h; 4) 25–29 h; 5) 30–34 h; 6) 25–39 h; 7) 40–45 h; and 8) >45 h
Research Question 2	Survey 2 Open-ended Item
	•Please describe how your participation in [*insert identified organization/society*] has contributed to your progress as you pursue your engineering degree

To determine participation in PEOs, we analyzed responses to the S2 item that asked students to mark which of the six types of listed “PEOs/societies” that they participated in during year 1. As shown in Table 2, in addition to the six types of societies/organizations that they could choose from, students also had the option to select an “other organizations/societies” category where they could write-in the name of other organizations/societies, or to select an answer of “none”. In reviewing the write-in responses to the “other organizations/societies,” students mainly identified engineering-related organizations/societies such as Robotics club, sororities, and fraternities. Therefore, we aggregated all of these responses as “PEOs”, acknowledging that student involvement or integration in any activity can contribute to persistence (Tinto 1998; Astin, 1999). A review of the responses to the “none” category revealed that some respondents participated in other activities, but in many cases, the description of these activities were vague or only tangentially related to science; therefore, we did not recode any of these responses.

To answer RQ1, we analyzed responses to items in S2-S5 that addressed enrollment status, reason for participation in PEOs, grade point average (GPA), employment status, and amount of study time. To answer RQ2, we analyzed responses to the open-ended item in S2 that asked students to describe how their participation in the PEOs that they identified contributed to their persistence in their engineering degree. We only included the responses of women and URM students who had graduated or persisted to their fifth year in our analysis. Although we asked students to think back to their first year in S2, which was administered in spring of the cohort’s second year, some students wrote about their experiences participating in these PEOs during their second year.

#### Statistical Analysis

All statistical analyses were performed using the SAS version eight software (SAS Institute Inc., 1999). We express as percentages women and URM students' participation in PEOs in the first year of their engineering program. To examine the extent to which participating in PEOs is associated with students’ persistence (RQ1), we performed logistic regression. This statistical method allows for modeling persistence, operationalized as a binary dependent variable (enrolled vs. not enrolled), as a function of participating in PEOs and other predictor variables without requiring that these variables are “normally distributed, linearly related, and have equal variance within each group” (Tabachnick and Fidell, 1996, p. 575). Because logistic regression assumes that the dependent variable varies as a function of the predictor variables, the logistic model calculates the probability of persisting, controlling for other variables, and expresses this probability as an adjusted odds ratio (AOR). Together with the associated *p*-values, the AORs are interpreted for the significant predictors in the models. The following five steps were followed to arrive at the model predicting persistence in year 2:

First, noting that our data set contained several potential covariates, the choice of variables to consider including in the model was guided by the literature review as detailed in our conceptual framework. The following four major classes of independent variables were included because of their potential theoretical and practical relationship to student persistence: 1) sociodemographic factors (i.e., being a woman vs. a man; being URM vs. white; being a URM woman vs. non-URM woman; and being at a minority serving institution [MSI] vs. non-MSI); 2) academic related factors (GPA [high ≥3.0, low ≤3.0]; amount of study time [high = spent >25 h on studies outside of class meeting times vs. less = spent <25 h]; how a student arrived at the decision to participate in a PEO [advised to participate by influencer or not, made own decision to participate or not]); 3) employment status (working fulltime, part-time, under federal work study, or summer/seasonal vs. not working at all); and 4) participation in PEO (women participating in PEOs vs. not; women in PWIs participating in PEOs vs. women in PWIs not participating in PEOs; URM students participating in a PEO vs. URM students not participating in a PEO; and being at a MSI and participating in a PEO vs. being at a MSI and not participating in a PEO). The dependent variable, persistence, was measured using enrollment status: students who indicated that they were still enrolled in their engineering major or had graduated with their engineering degree in S2-S5 were coded as “persisters” whereas those who indicated that they had switched to a non-engineering major or had graduated with a non-engineering degree were coded as “non-persisters.”

Second, to aid the selection of variables to examine closely, we ran a correlation procedure. Whenever a set of variables were highly correlated, only one variable in each case was considered for further analysis so as to avoid multicollinearity. Third, we examined the bivariate relationship between persistence and each of these variables, using chi square test (*p* value of <0.05 was considered to be statistically significant). Fourth, beginning with the effect of sociodemographic factors and before arriving at the final model in year 2, we considered several models (not presented here), each time retaining only statistically significant covariates in the succeeding models. Finally, the overall fit of models tested were evaluated using the −2 log likelihood [−2LL] statistic (for nested models) and the Akaike information criterion (AIC) (Akaike, 1973) for non-nested models. These statistics are interpreted as follows: the smaller the −2LL, the better is the fit, and an AIC value closer to zero represents a better fit. Complementing the use of AIC and −2LL for assessing model fit, we used the likelihood ratio chi-square, a statistic that shows whether the model fits significantly than an empty model (i.e. a model not including any predictors). Once the best fitting model for year two was obtained, the analysis was repeated for year three–five so as to aid comparison of findings based on similar set of covariates.

#### Thematic Analysis

To determine how women and URM students acquire social capital, thematic analysis was performed on the responses to the open-ended S2 items that asked how participation in the PEOs, with a focus on SWE, NSBE, and SHPE, contributed to their persistence. Three members of the qualitative team coded the responses independently. After the first team member coded the open-ended data independently, the second team member reviewed the coded data, developed the codebook, and then coded the data independently. The third team member coded the data guided by the codebook. The first team member then reviewed all the coded data, the codebook, and finalized the coding based on the commonality of the themes coded by team members. The second and third team members then reviewed the coded data and codebook together (the first team member was unavailable). They discussed disagreements over coding and reached consensus by either assigning a response to the same code or doubling coding it. They then identified themes from the coded data by noting key terms that were repeated about participation in SWE, NSBE, and SHPE such as “helped,” “gave,” “community,” “opportunity,” and “friendships” (Braun and Clarke, 2006; Bazeley & Jackson, 2013). Along with the key terms, student descriptions of the social capital acquired from SWE, NSBE, and SHPE were grouped together by type. The analyses were based on frequency, patterns, and “keyness” (i.e., the extent to which the data captured concepts that are essentially related to our research questions).

## Results

### Participant Characteristics

The gender and race/ethnicity of students who responded to each survey are presented in Table 3. Women are represented at a higher proportion in our sample than found nationally. They represented more than a third of the respondents in all five surveys (S1 = 35%; S2 = 35%, S3 = 35%, S4 = 35%, S5 = 36%). In 2015–2016 women were 19.2% (*n* = 104,033) of the 541,705 students enrolled in engineering (and engineering-related) majors (NSF, 2017). All women, including those who are not URM students (i.e., white, Asian), are included in analyses of women’s responses. Likewise, URM students comprised a higher proportion of our sample (S1 = 32%; S2 = 32%, S3 = 32%, S4 = 31%, S5 = 32%) than they did of the national population of engineering undergraduates, where they comprise 16.5% (89,616) of the total of 541,705 (NSF, 2017). The likely reason for the high proportion of URM students is that we recruited participants from one HBCU and three HSIs. Another possible reason for the overrepresentation of women and URM students in our study is that the study’s informed consent form explained that the goal of the study was to understand women and URM students’ experiences in engineering. This focus may have encouraged women and URM students' responses and potentially discouraged responses from those not identifying as such. NSF does not count foreign nationals as URM students, but we included them in the reported race/ethnic groups.

**TABLE 3:** Respondent Race/Ethnicity by Gender for Surveys 1, 2, 3, 4, and 5.

Race/Ethnicity	Survey 1		Survey 2		Survey 3		Survey 4		Survey 5	
	Men	Women	Men	Women	Men	Women	Men	Women	Men	Women
American indian	5	0	5	0	4	0	4	0	4	0
Asian	131	72	131	72	121	55	108	50	97	47
Black	40	26	40	26	26	16	25	16	20	16
Latinx	224	106	224	106	186	85	173	80	164	75
Middle eastern	13	4	13	4	11	4	11	4	9	3
Nat. Hawaiian	0	1	0	1	0	1	0	1	0	1
Other	7	15	7	15	6	14	6	14	4	14
White	393	215	393	215	301	173	286	169	267	162
Total	813	439	813	439	655	348	613	334	565	318

### Participation in Professional Engineering Organizations

Forty-nine percent (*n* = 612) of the 1,252 respondents to S2 indicated they had participated in a PEO during their first year in their engineering program. Of these 612 students, the highest proportion participated in industry-specific PEOs (43%), women-focused PEOs (31%), race/ethnicity-focused PEOs (20%), and other organizations/societies (22%), with honor societies having the lowest proportion of students (12%) (Table 4). A large percentage of women (42%) participated in women-focused PEOs whereas a quarter of URM students (27%) participated in race/ethnicity-focused PEOs. Of all the URM students in our sample, 55% of Blacks and 22% of Latinx participated in race/ethnicity-focused PEOs. Regarding respondents who were both women and URM, 69% of Black women and 17% of Latina women participated in race/ethnicity-focused PEOs. In contrast, half of white women (50%) and half (51%) of Asian women participated in women-focused PEOs.

**TABLE 4:** Participation in Professional Engineering Organizations by Category, Gender, and Race/Ethnicity Intersection (Survey 2).

Category of Professional Engineering Organization	Total Students	Women	Under-represented minorities	Black	Hispanic	Black women	Latina women	White women	Asian women
	N	*n*	*n*	*n*	*n*	*n*	*n*	*n*	*n*
Industry-specific	264	90	95	9	84	1	25	46	11
Women-focused	190	183	33	9	24	9	20	108	37
Race/Ethnicity-focused	124	42	108	36	72	18	18	5	1
Other	136	53	25	2	19	4	8	30	9
Honor society	76	20	19	3	16	1	6	12	1

### Persistence and Participation in Professional Engineering Organizations (RQ1)

Holding constant other factors, URM students participating in PEOs have increased odds of persisting to the second year than URM students not participating in PEOs have (adjusted odds ratio [AOR] = 2.18, CI: 1.09, 4.37). Table 5 shows the prediction of enrollment across the 5 years and independent variables. However, women’s participation in PEOs, women in PWI participation in PEOs, or attending a MSI and participating in PEO, are each not associated with persistence, ceteris paribus.

**TABLE 5:** Prediction of Enrollment in Year 2, Year 3, Year 4, and Year 5.

Variable	Year 2	Year 3	Year 4	Year 5
	AOR (95% CI)	AOR (95% CI)	AOR (95% CI)	AOR (95% CI)
Sociodemographic				
Women	1.49 (0.94, 2.36)	3.09 (1.19, 8.03)^a^	1.17 (0.82, 1.65)	1.13 (0.81, 1.58)
Underrepresented minorities (URM)	0.67 (0.39, 1.13)	1.42 (0.46, 4.41)	0.73 (0.47, 1.14)	0.79 (0.51, 1.22)
URM women	0.64 (0.33, 1.26)	0.82 (0.15, 4.36)	1.01 (0.61, 1.69)	1.01 (0.62, 1.66)
Being at a minority serving institution (MSI)	3.60 (1.92, 6.76)^a^	2.87 (0.79, 10.5)	1.57 (0.97, 2.55)	1.29 (0.81, 2.07)
Academic-related factors				
Current grade point average (high: ≥3.0; low: <3.0)	4.14 (3.30, 5.19)^a^	9.08 (5.99, 13.8)^a^	2.10 (1.76, 2.50)^a^	1.43 (1.21, 1.69)^a^
More study time vs. less study time	1.52 (1.14, 2.04)^a^	2.01 (1.14, 3.54)^a^	1.02 (0.83, 1.26)	0.96 (0.79, 1.17)
Decision to participate				
Advised to participate by alter/influencer (Yes = 1, No = 0)	2.76 (1.40, 5.43)^a^	1.17 (0.46, 2.97)	1.28 (0.86, 1.90)	1.07 (0.74, 1.54)
Own decision to participate (Yes = 1, No = 0)	2.96 (1.55, 5.66)^a^	2.05 (0.76, 5.51)	1.37 (0.94, 2.01)	1.16 (0.81, 1.65)
Employment				
Worked vs. unemployed	1.45 (1.13, 1.87)^a^	2.78 (1.77, 4.36)^a^	0.85 (0.70, 1.04)	0.73 (0.61, 0.88)^a^
Participation in PEOs				
Women participating vs. women not participating	0.50 (0.13, 1.84)	0.19 (0.02, 1.50)	0.66 (0.29, 1.52)	0.61 (0.27, 1.34)
Women in PWI participating vs. women in PWI not participating	1.52 (0.40, 5.75)	9.47 (0.99, 90.5)	1.65 (0.71, 3.86)	1.85 (0.82, 4.15)
URM students participating vs. URM student not participating	2.18 (1.09, 4.37)^a^	1.23 (0.28, 5.40)	1.09 (0.63, 1.88)	1.19 (0.70, 2.02)
Attending a MSI and participating vs. attending in MSI and not participating	0.78 (0.25, 2.48)	0.64 (0.10, 4.29)	1.20 (0.57, 2.52)	1.31 (0.64, 2.65)
Model fit statistics				
Akaike information criterion (AIC)	1,365.597	494.82	2,131.959	2,272.481
-2 loglikelihood (-2 L L)	1,339.597	468.82	2,105.959	2,246.481

a *p* ≤ .05; AOR (Adjusted Odds Ratio) indicate the likelihood of persisting to a given year as opposed to not persisting for a unit increase in a given independent variable, holding constant other variables in the model; AOR values greater than 1.0 denote a greater likelihood of a student persisting than not persisting for each unit increase in the independent variable; and AOR values less than 1.0 denote a lower likelihood of a student persisting than not persisting for each unit increase in the independent variable.

Overall, women are more likely than men to persist in third year (AOR = 3.09, CI: 1.19, 8.03) but not fourth or fifth year. Being an URM or URM woman is not associated with persistence from years two to five. However, being at a MSI is associated with increased odds of persistence in second year (AOR = 3.60, CI: 1.92, 6.76), but not years three to five. Other factors associated with increased odds of persistence include having a high GPA as opposed to a low GPA (second to fifth year); spending more time studying as opposed to less time (second and third year); and being advised to participate in a PEO by an influencer or deciding by oneself to participate (second year only) as opposed to not being advised to participate by influencer or not deciding by oneself, respectively. Working, as opposed to being unemployed, was associated with increased odds of persistence in second and third years but decreased odds of persistence in fourth year.

As noted earlier, some students participated in more than one PEO. For example, 29% of the women who participated in women-focused PEOs and 26% of the URM students who participated race/ethnicity-focused PEOs also participated in industry-specific PEOs. We examined whether participation in multiple PEOs vs. a single PEO made a difference in persistence but found that it did not. Thus, to present a more parsimonious model, we did not include this variable among predictors in our logistic regression.

### Social Capital From Professional Engineering Organizations (RQ2)

#### Underrepresented and Minoritized Students in Professional Engineering Organizations

Thematic analysis of URM students’ open-ended responses provides insight into the ways the acquired social capital from PEOs contributed to their persistence. Black and Latinx students described the social capital they accessed and activated due to their participation in NSBE and SHPE. The three primary forms of social capital were 1) academic and social integration through academic support, such as developing time management skills and tutoring, as well as social networking, such as meeting other students and engineers of color some of whom become friends and mentors; 2) connecting with industry internships and employment opportunities though attendance at national conferences; and 3) professional resources for career development such as improving leadership skills, resume writing, and interview skills.

Among the URM students who participated in PEOs in their first year, Black students (15 women and nine men) described how NSBE contributed to their success. The three Black students (1 woman and two men) explained that their participation in industry-specific PEOs reinforced their decision to pursue their engineering major and provided career opportunities and other activities through networking. Four Black women who also participated in SWE stated that through social networking they attended professional conferences and gained industry contacts and internship opportunities as well as a mentor. Two other Black women felt they did not gain anything by participating and one decided to stop participating in SWE and chose to focus her participation in NSBE instead.

Black students stressed that participating in NSBE reduced their isolation at PWIs. By participating in NSBE, they became part of a culturally familiar community whose membership was comprised of Black students and engineers who had similar engineering experiences. A Black man at a PWI wrote, “I feel that [NSBE] has connected me to other Black engineers and allowed me to remain close with some of the people from my [bridge program] cohort.” A Black woman at a PWI agreed, “[NSBE] has helped me connect with people who are like me going through the same things.” Several Black students explained that the social capital they accessed and activated by participating in NSBE in their first year continued to benefit them in their second year. A Black woman at a PWI reported, “in my first year of college [NSBE] introduced me to the importance of setting career goals. Now I am a sophomore … , I hold an elected position and will be attending the national conference in hopes of obtaining an internship or co-op. My academic performance has also improved.” Another Black woman at a PWI explained:

NSBE has helped me establish another family in the College of Engineering. I have gotten a lot of help preparing for career fairs and things of that nature from my peers in this organization. NSBE has also given me an opportunity to grow more as a person and learn a lot about leadership as I have moved up in the organization. It has provided me with tutors and great relationships that will help me well after I graduate.

A Black man at a PWI concurred, “NSBE gives me tools to develop time management skills, resume skills, elevator pitches and much more.”

Latinx students described how industry-specific PEOs (45 men and 11 women) and SHPE (43 men and 11 women) contributed to their persistence as engineering majors. For Latinx students, industry-specific PEOs expanded their knowledge about their disciplines, provided information about the various careers available with their majors, as well as delivered critical sources of networking for internship and employment opportunities. For example, a Latino man at a PWI wrote:

Being able to see the extent of the electrical engineering student body participating in IEEE [Institute of Electrical and Electronics Engineers] was inspiring and made me more eager to continue my studies. The topics covered in society meetings and events were slightly above my understanding, but made me more interested in a variety of EE topics.

Latinx students reported that SHPE provided access to professional resources and provided a familiar cultural environment where they could network and make friends with other Latinx students and engineers. A Latinx man at a PWI stressed the importance of cultural familiarity, “SHPE was a great way to maintain a sense of home while at university. Having a Hispanic culture with the engineering world made all the difference.” Explaining the value of the relationships formed through SHPE, a Latina woman at a PWI shared:

I began by rarely attending meetings to now being an active member. Attending the SHPE Conference this year has been one of the best decisions I have made this year by far. I collaborate with incredibly intelligent people from similar backgrounds who serve as role models and friends.

In addition to cultural familiarity, Latinx students explained how SHPE helped them develop professional skills. For example, a Latinx man at an HSI stated, “[SHPE] helped me develop my skills in time management and served as an excellent opportunity to meet people that already had experienced different types of situations at the university and hear how they solved them.” A Latinx man at a PWI explained the value of the professional resources:

Participation in SHPE has given me a lot of resources to be successful, such as resume critiques, private career fairs with only our organization. Furthermore, it allowed me to connect with students in my classes who I have become friends with and can rely on for help when I need it.

A Latina woman at an HSI concurred:

SHPE has really helped me develop my leadership skills … Second year I started getting really involved with the community. Not only [did] I organize many activities during the semester, but I also got the opportunity to travel to Washington, DC for the National Science Bowl. It really has helped me with my communication skills as I approach recruiters, as well as create a resume and perform a good interview.

Overall, the responses to the open-ended responses reveal how URM students, and in particular, Black and Latinx students who represented the numerical majority of URM students in our sample, benefitted from participating in PEOs.

#### Women in Women-Focused Professional Engineering Organizations

Although we found no statistically significant association between women’s participation in PEOs and persistence, women who participated in SWE reported acquiring social capital similar to that acquired by URM students who participated in race/ethnicity-focused PEOs. For example, women emphasized that participating in SWE reduced their gender isolation. They also noted that participating in SWE increased their confidence in their knowledge and abilities to succeed in their engineering programs and pursue successful careers as engineers.

Women reported socializing and networking with other women students (some senior to them), women engineering faculty, and professional women engineers with established careers at SWE events who talked about what it was like to be a practicing engineer. A white woman at a PWI stated, “having a professional society with women experiencing the same things as me really helped me gain confidence in my first year as an engineering student.” A white woman at a PWI agreed, explaining:

My participation in SWE has contributed to my progress to pursue my engineering degree by reminding me that even though engineering classes and events are dominated by males, females can still be loud and proud and make a difference in the engineering field. It is hard sometimes, but it is possible.

A white woman at a PWI concurred:

It is encouraging to realize there are more women in engineering than you might think by just attending class. SWE is a great way to make friends and meet study-buddies with other girls when your classes are guy-heavy. They also have a mentorship program.

Through the relationships and interactions with other women in SWE, women form a community that provides emotional support and a sense of belonging. These relationships also provide social capital by conveying critical insider knowledge about succeeding and persisting as a woman in engineering.

Describing the benefits of social networking, a white woman at a PWI stated, “as a member of SWE, I have attended various community events, received a mentor to help guide me through my degree, and have been to various industry networking events.” The friendships with peers in SWE also provided invaluable academic and emotional support as noted by a white woman at a PWI:

I currently hold an officer position and the friendships I have made through this Organization [SWE] are very rare. We study together. We hang out together. I have formed an amazing group of friends who all push each other to keep going. We all understand each other’s struggles.

An Asian woman at PWI agreed about the beneficial resources available through SWE:

I got to listen to speakers [who] come from companies I respect and learn what they look for in a student. Being a first year meant that I had plenty of space to shape the type of student I wanted to become.

For the women in our study, SWE was a critical source of social capital that, when activated, contributed to their persistence in a field where women students and faculty were few and far between.

## Summary of Results

In sum, we found that: 1) URM students participating in PEOs (including engineering-related activities) were more likely to persist to the second year than URM students not participating in them, and 2) women and URM students report that they acquire social capital from their participation in gender and race/ethnicity-focused PEOs through social networking, reducing gender isolation, reducing race/ethnic isolation, and accessing professional resources.

## Discussion

Our results align with (Astin’s 1999) student involvement theory and (Tinto’s 1998) theory of student departure, which posit that higher levels of student involvement and integration in campus life lead to improved student learning outcomes and persistence. Not determined by other studies, we found a significant relationship between URM students’ participation in PEOs (including engineering-related activities) and their persistence to the second year compared to their third to fifth year. Additional research is required to understand this phenomenon. However, this finding suggests that establishing early connections to engineering may influence URM students understandings of expectation in ways that the diminishes the differences that may appear between them and their colleagues in later years.

Our analysis of the open-ended items provides additional evidence supporting prior research by highlighting a critical source of social capital for URM students who are traditionally excluded from engineering. Consistent with Martin and colleague’s (2016) assertion, we found that race/ethnicity-focused PEOs serve as cultural enclaves on white PWI campuses. In addition to providing URM students with social capital, race/ethnicity-focused PEOs are also welcoming culturally familiar environments that cushion the cultural disruption of attending PWIs (Tierney, 1992). Consistent with Daily, Eugene, and Prewitt’s (2007), we also found that NSBE is a critical source of social capital through its social networks because it creates a culture that contributes to academic achievement and retention of Black students in engineering. We extend this finding by reporting how students benefits from participating in race/ethnicity-focused PEOs to persistence.

Although we did not find a statistically significant relationship between participating in gender-and race/ethnicity-focused PEOs and engineering persistence, our qualitative findings are consistent with previous qualitative studies that found that URM students who participated in NSBE and SHPE and women who participated in SWE reported acquiring social capital that benefited their persistence (Daily et al., 2007; Goodman et al., 2002; Martin, et al., 2016; Martin et al., 2016; Ross and McGrade, 2016; Revelo and Baber, 2018; Strauss and Terenzi, 2007). Black students reported that NSBE was the primary source of social capital from PEOs while Latinx students identified both industry-specific PEOs and SHPE as their primary sources of social capital. Further, participating in race/ethnicity-focused PEOs reduces URM students’ isolation, which is often exacerbated for URM women because of their dual minority identities related to gender and race/ethnicity. Participation in SWE increased the confidence of women who persisted to their fifth year in addition to reducing their gender isolation. This finding supports (Cech et al., 2011)’ (2011, p. 658) claim that confidence is “important to students” behavioral and intentional persistence.”

Our finding that URM students and women acquire social capital from PEOs, particularly women- and race/ethnicity-focused PEOs, is consistent with and reinforces (Lin’s 2001) assertion that social capital is rooted in relationships and interactions with individuals engaging in agency for their benefit. Such relationships allow members to purposively activate social capital and receive it in “routine exchanges” (Martin, 2015, *p*.1180). As noted earlier, we refer to the social capital (i.e., the personal relationships/ties, social networks, and professional resources) attained through these organizations as participatory social capital (Author, 2020a). Researchers (Lin, 2001) assert that individuals gain social capital from the people directly in their network, and our findings indicate that students who enter an unfamiliar environment can establish relationships and gain social capital as they participate in organizations designed to provide this level of support, thereby extending their network and access to additional social capital.

Our findings also align with Martin and colleagues’ (2016) that found that NSBE and SHPE provide ample opportunities for establishing mentoring and role model relationships and creating tight, “family like” bonds that last throughout students’ college careers and beyond. For URM engineering students, the one key benefit of participating in race/ethnicity-focused PEOs is the opportunity to gain insider knowledge, a form of social capital. We found that the majority of students who joined race/ethnicity-focused PEOs did so because they were advised to do so by the most influential person in their social network.

### Limitations

We acknowledge that capturing social capital retrospectively has the potential for recall bias when students are asked to “think back” to the prior year about their participation in SWE, NSBE, and SHPE. This retrospective approach examined whether levels of social capital at college entry change or remain stable over the first four years of an engineering program. However, given the short duration of time lapse (∼1 year since the end of their first year), we expect their recall to be reliable overall.

Our sample size limited some intersectional analyses, and some variables were linear combinations of others and thus were not included in the list of predictors. Surveys 2 (S2) to S5 were administered to students who reported that they were still enrolled as engineering majors in the prior survey. As expected, the sample decreased with each subsequent administration of the survey. Despite this, 60% of the initial S1 engineering respondents (*n* = 2,102) completed all of the surveys (*n* = 1,252).

Finally, S1 was conducted in the spring 2015 semester when students were enrolled in the second semester of their first year. Because of this, our sample does not include any students who may have switched out of engineering after their first semester of enrollment in fall 2014. Therefore, our sample may underrepresent the percentage of switchers. Hence, we encourage researchers who may engage in similar studies to consider the potential impact on their data collection efforts. Specifically, obtaining information from students in their first semester of college enrollment may provide insights about other factors that influence their attrition.

### Implications for Practice

Our results provide evidence to warrant further investment in race/ethnicity-focused PEOs such as NSBE and SHPE to aid URM students and ultimately promote diversity, equity, and inclusion in the STEM workforce. Specifically, advisors should encourage URM students to join and participate in PEOs beginning in their first year given the influence of such involvement to persistence. During their earlier years in the program, many engineering students are at highest risk for switching out (Meyer and Marx, 2014). Engineering programs should also partner with PEOs to 1) develop strategies to transform their culture so it becomes welcoming to women and URM students, and 2) sponsor campus events/activities that embrace and promote cultural diversity as a strength that can foster a sense of community for women and URM students (Tierney, 1992; Martin et al., 2016). We support and extend Martin and colleagues’ (2016) call to action “for all engineering faculty—majority and underrepresented—to recognize the value of ethnic student organizations. . . and to explicitly support these organizations.” Administrators can also financially support student participation in these PEOs, by paying member fees, funding travel to their professional conference, and the like. Faculty can collaborate with PEOs to provide research opportunities and career-oriented information. Doing so will support social capital development for women and URM students and, ultimately, contribute to their persistence. Additionally, all PEOs, not just those that are women- and race/ethnicity focused, should prioritize ensuring that they are welcoming and inclusive to women and ethnic minority students (Campbell-Montalvo et al., 2020).

## Data Availability

The raw data supporting the conclusions of this article will be made available by the authors, without undue reservation.
